# Efficacy and safety of novel anticoagulant therapies in patients with chronic kidney disease—a systematic review and meta-analysis

**DOI:** 10.1007/s40620-024-02130-3

**Published:** 2024-11-29

**Authors:** Ernesto Calderon Martinez, Camila Sanchez Cruz, Edna Y. Diarte Acosta, Daniel Alejandro Aguirre Cano, Ana Maria Espinosa, Diana Othón Martínez, Flor Furman, Sebastian Obando Vera

**Affiliations:** 1 Universidad Nacional Autónoma de México, Ciudad de Mexico, México; 2 Universidad Autónoma de Sinaloa, Sinaloa, México; 3 Universidad de Monterrey, Monterrey, México; 4 Univesidad Nacional de Loja, Loja, Ecuador; 5 University of Texas RGV, Edinburg, TX USA; 6 Universidad de Buenos Aires, Buenos Aires, Argentina; 7 Universidad Catolica de Santa Maria, Arequipa, Perú

**Keywords:** Chronic kidney disease, Anticoagulation, Direct oral anticoagulants, Vitamin K antagonists

## Abstract

**Background:**

Chronic Kidney Disease (CKD) significantly increases the risk of cardiovascular diseases, including atrial fibrillation, which usually requires anticoagulant therapy. The effectiveness and safety of direct oral anticoagulants compared to vitamin K antagonists in patients with CKD remain insufficiently studied, particularly in the more advanced stages.

**Methods:**

This systematic review, registered in PROSPERO (CRD42023410192), adhered to PRISMA guidelines and included randomized clinical trials and cohort studies comparing direct oral anticoagulants and vitamin K antagonists in CKD patients. Major databases were searched, and studies were selected based on strict inclusion criteria. A meta-analysis was performed using random-effects models.

**Results:**

Twenty-three studies with a total of 465,673 CKD patients were included. Direct oral anticoagulants showed a significant reduction in major bleeding events compared to vitamin K antagonists (Relative Risk [RR] = 0.62, 95% Confidence Interval: 0.49–0.79, *p* < 0.01) and a non-significant trend toward reducing thromboembolic events (RR = 0.69, 95% Confidence Interval: 0.43–1.14, *p* = 0.11). Furthermore, direct oral anticoagulants were associated with a significant reduction in all-cause mortality (RR = 0.63, 95% Confidence Interval: 0.43–0.91, *p* = 0.02).

**Conclusion:**

Direct oral anticoagulants may offer a safe alternative to vitamin K antagonists in CKD patients, particularly in terms of reducing bleeding risks and potentially improving survival. However, their role in preventing thromboembolic events remains uncertain, highlighting the need for further research, especially in patients with advanced CKD and kidney failure.

**Graphical abstract:**

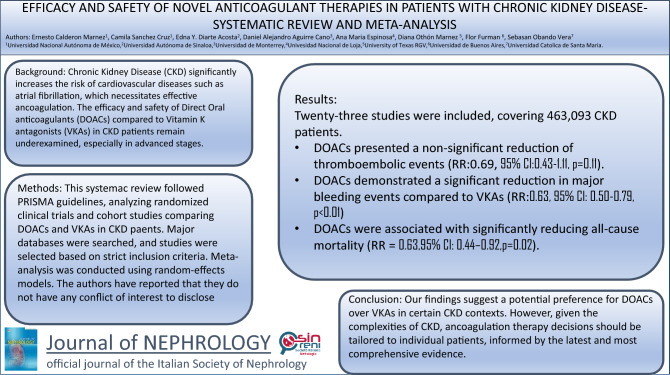

**Supplementary Information:**

The online version contains supplementary material available at 10.1007/s40620-024-02130-3.

## Introduction

Chronic kidney disease (CKD) is a condition characterized by irreversible loss of kidney function. The guidelines define CKD as either kidney damage or a glomerular filtration rate (GFR) of less than 60 mL/min/1.73 m^2^ for at least three months. It can arise from chronic conditions such as diabetes mellitus or hypertension [1, 2]. Patients with CKD have a high prevalence of cardiovascular diseases. One of the most common cardiovascular diseases in patients with CKD is atrial fibrillation [2–4]. CKD predisposes to atrial fibrillation because of increased arrhythmogenic and inflammatory markers, left atrial enlargement, and diastolic dysfunction. Myocardial fibrosis is likewise commonly reported [1, 77]. Additionally, increased potassium levels can cause electrical disturbances [1]. Thromboembolic disease and heart failure are feared complications in patients with CKD. The two anticoagulation approaches are Vitamin K antagonists and the novel anticoagulants known as Direct Oral Anticoagulants (DOACs) [5]. Direct oral anticoagulants were introduced to the general population around 2010 [6]. Kidney function is crucial for the clearance of direct oral anticoagulants. Dabigatran is a direct thrombin inhibitor with around 80% kidney clearance. Direct factor Xa inhibitors (Edoxaban, Rivaroxaban, Apixaban) have lower kidney excretion, ranging from 30 to 50% [2]. In patients with normal kidney function, direct oral anticoagulants are a safe alternative for anticoagulation; the same holds true in patients with chronic kidney disease and atrial fibrillation with an intermediate risk of embolization per CHA2DS2-VASc (7,8) due to their decreased drug interactions, faster metabolism, and lack of need to monitor coagulation. These advantages are shared by patients with creatinine clearance of > 25–30 mL/min [4, 7–9]. Further investigation is required to assess the safety of direct oral anticoagulants in patients with stage 4 CKD and kidney failure.

## Methods

This review used the Preferred Reporting Items for Systematic Reviews and Meta-Analysis (PRISMA) 2020 guidelines and recommendations from the Cochrane Handbook [10, 11].

### Search strategy

The databases PubMed, EMBASE, Cochrane, Web of Science, LILACS, Clinicaltrials.gov, and International Clinical Trials Registry Platform were systematically searched on 10th January, 2024 to identify pertinent articles published from the inception of these databases. Combinations of search terms were utilized, including: ‘’anticoagulants’’, ‘’Vitamin K’’, and ‘’Chronic Kidney disease’’, for the entire search strategy per database, see supplementary material (Online Resource 1–7). Our protocol was registered on PROSPERO under the ID: CRD42023410192.

### Selection of studies

We included randomized clinical trials, case–control studies, and prospective or retrospective cohort studies from 2013 to 2023 to ensure inclusion of the strictest and most up-to-date data available in English and Spanish. Studies analyzed in previous meta-analyses were also considered. Case reports, case series, population-based cross-sectional studies, cohort studies, dissertations, book chapters, protocol articles, reviews, news articles, conference abstracts, letters to the editor, editorials, and comment publications were excluded. Studies with no clear description of their methodology, duplicates, and incomplete data were also excluded.

### Type of participants

Patients with CKD above 18 years of age were included. Patients with hemophilia with low levels of either factor VIII or factor XI were excluded. CKD was defined as per the KDOQI guidelines [12].

Studies comparing vitamin K antagonists and direct oral anticoagulants for anticoagulation therapy in patients with CKD were selected.

The main outcomes were the incidence of thromboembolic events, major bleeding events, and all-cause mortality. Data were stratified according to the most widely used risk score (CHA2DS2-VASc).

The search results were imported into Rayyan [13], where duplicates were removed, and relevant inclusion and exclusion keywords were added to make the screening process easier and more efficient. Following an initial screening based on the title and abstract, two reviewers (FF and DO) independently selected trials for inclusion in this review using predetermined inclusion and exclusion criteria. Keywords highlighted inclusion and exclusion criteria-related words on Rayyan [13] (Online Resources 8 and 9). Subsequently, a full-text analysis was conducted, with two reviewers (FF and DO) independently selecting trials for inclusion in this review using predetermined inclusion and exclusion criteria. Disagreements about the inclusion of studies in any of these steps were resolved through consensus and consultation with a third review author (ECM). We contacted the authors of articles that lacked full text by email. If there was no reply, we excluded those studies from the analysis. Once the screening process was completed, data were extracted from the eligible studies into a standardized Microsoft Excel spreadsheet by two independent reviewers. Two reviewers extracted data independently. Discrepancies in data extraction were resolved through consensus and by consulting a third reviewer (ECM).

### Quality assessment

We evaluated the studies using the criteria outlined in the Cochrane Handbook [14]. To assess the quality of studies included in the systematic review and meta-analysis, we applied the Cochrane Risk of Bias 2.0 tool, which examines potential bias in domains including selection, performance, detection, reporting, attrition, and other sources of bias for randomized controlled trials. Additionally, for case–control and prospective, retrospective cohort studies included in the review, we employed the Newcastle–Ottawa Scale to assess the risk of bias [15, 16]. Two independent reviewers evaluated the risk of bias in each study, considering the specific criteria and guidelines provided by the respective tools. Any discrepancies between the reviewers were resolved by consulting with a third, blinded reviewer as needed. The methodological components of the trials and case–control studies were assessed as having a low, high, or unclear risk of bias by the Cochrane Handbook for Systematic Reviews of Interventions and the Newcastle–Ottawa Scale guidelines, respectively. The summary of findings table presented details of any downgrading or upgrading of the quality of evidence, providing transparency and explanations for the assessment of bias in each included study.

### Statistical analysis

Meta-analysis was performed using the R Software version 4.3.3 (R Core Team, 2024) to calculate the effect size [17]. Effect sizes were presented as Relative Risk (RR) with 95% Confidence Intervals (CI). The random-effects model was used for pooling analysis to compensate for the heterogeneity of statistics [18, 19]. In this regard, ≥ 50% and ≥ 75% indicated substantial and high heterogenity, respectively. For all outcomes, sensitivity analyses according to the leave-one-out method were performed to determine the influence of individual studies on the overall effect. Egger’s regression test examined publication bias when 10 or more reports with the same outcome were available. Whenever possible, subgroup analyses were performed for primary outcomes, p-values < 0.05 were considered statistically significant [19].

## Results

**Fig. 1:**
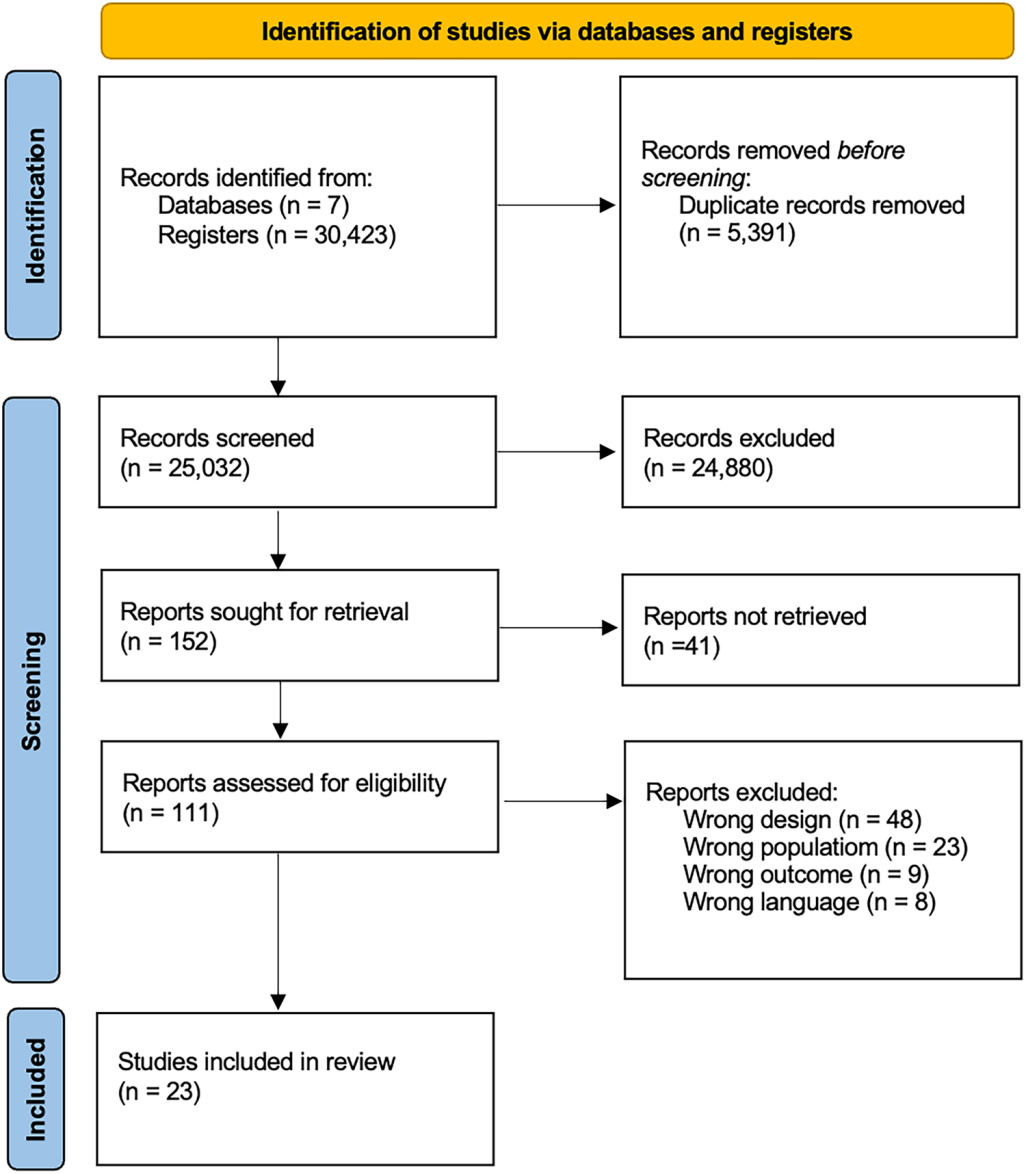
Prisma Flow Chart

Initially, a systematic search of the aforementioned databases provided 30,423 citations. After removing duplicates, 25,032 articles were screened by title/abstract, excluding irrelevant ones. The 152 remaining studies were scrutinized across the full text and 129 were discarded. Thus, 23 studies met the inclusion criteria and were included in the qualitative review, while only 19 were included in the quantitative analysis. A summary of the study selection process is presented in Fig. 1 according to the PRISMA guidelines [10, 11, 20].

### Systematic review results

**Fig. 2:**
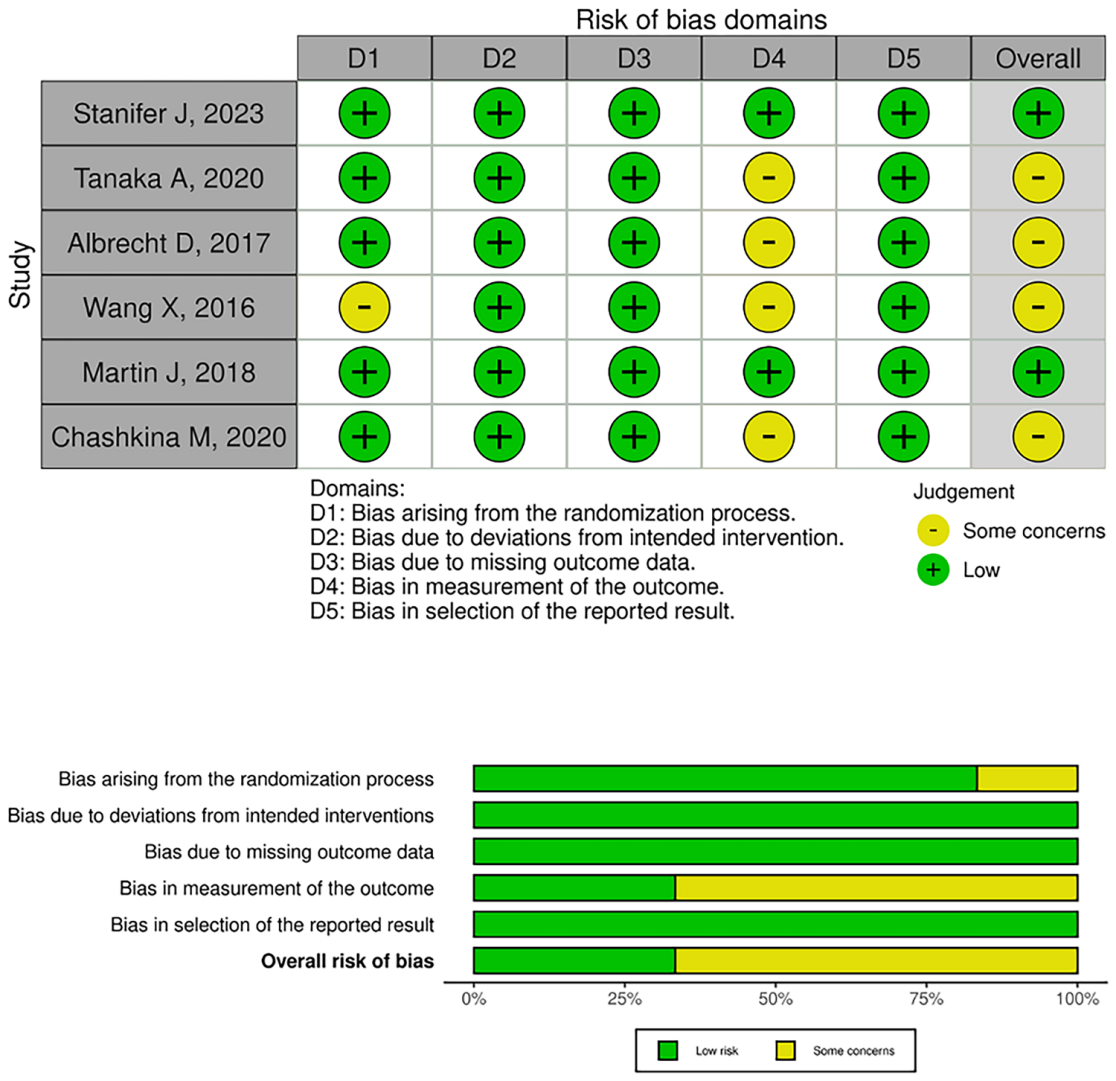
Traffic light plot of risk of bias assessment for each included article

The primary outcomes analyzed from the studies were thromboembolic, bleeding, and all-cause mortality events. The included studies were conducted across a broad geographic range, including the United States (57.14%), Taiwan (14.29%), Germany (9.52%), Japan (4.76%), Russia (4.76%), Israel (4.76%), South Korea (4.76%), and Italy (4.76%). An aggregate of 21 articles were reviewed, with a total sample size of 465,673 participants, encompassing patients with different stages of CKD. The study pool employed vitamin K antagonists and direct oral anticoagulants. These studies ranged from a few months to a couple of years duration. Furthermore, almost all the interventions showed that direct oral anticoagulants decrease major bleeding events and are associated with a lower risk of all-cause mortality. Of the 21 articles reviewed, 18 were found to have a low risk of bias, 3 with some concerns, and 0 with a high risk of bias [3, 21–52]. Figure 2 and Table 1 summarize the information on the risk of bias for each study and domain assessed. Table 2 shows the most important characteristics of the studies included.

**Table 1:** Newcastle Ottawa Scale for included articles

Author, year	Selection	Comparability	Outcome	Total	Quality
Wetmore, 2021	4	1	3	8	Good Quality
Herndon, 2020	4	1	3	8	Good Quality
Sakai, 2022	4	1	3	8	Good Quality
Chang S, 2019	4	1	3	8	Good Quality
Sy, 2021	4	2	3	9	Good Quality
Yao, 2020	4	2	3	9	Good Quality
Lee, 2021	4	1	3	8	Good Quality
Fu, 2021	4	1	3	8	Good Quality
Gurevitz, 2021	4	1	3	8	Good Quality
Bonnemeier H, 2019	4	1	3	8	Good Quality
Lee K, 2015	3	1	3	7	Good Quality
Ahuja, 2021	4	1	3	8	Good Quality
Laugesen E, 2019	4	0	3	7	Good Quality
Shin, 2018	4	0	3	7	Good Quality
Coleman, 2019	4	0	3	7	Good Quality
Weir, 2020	4	0	3	7	Good Quality
Di Lullo, 2018	4	2	3	9	Good Quality
Schafer, 2018	4	1	3	8	Good Quality
Makani, 2020	4	1	3	8	Good Quality
Sarratt, 2017	4	0	3	7	Good Quality
Kee K, 2023	4	1	3	8	Good Quality
Hsu, C, 2023	3	1	3	7	Good Quality

*Own authorship

**Table 2:** General Outcomes

Author(s)	Year	Country	Study design	Total sample size	Population characteristics	Specific intervention	Specific comparator	Key points
Wetmore J	2021	United States	Retrospective Cohort	12,816	Patients with CKD stages 3, 4, or 5 and incident AF	Apixaban	Warfarin (INR goal range: 2–3)	Apixaban, compared with warfarin: Lower risk of CKD stage progression
Herndon K	2020	United States	Retrospective Cohort	111	Veterans with CKD stage 4 and 5	Apixaban (2.5 mg or 5 mg twice daily)	Warfarin (INR goal range: 2–3)	No difference in major bleeding between apixaban and warfarin, but statistically significant increases in rates of composite bleeding and minor bleeding with apixaban
Sakai T	2022	Japan	Prospective Cohort	329	Elderly patients (> 65 years) with NVAF, HF, and renal dysfunction	DOACs not specified	VKAs not specified	DOAC therapy associated with lower risk of all-cause mortality in elderly HF patients with AF and renal dysfunction compared with VKA therapy
Stanifer J	2020	United States	Randomized Controlled Trial	269	Patients with AF and advanced CKD stages 4 and 5	Apixaban	Warfarin (INR goal range: 2–3)	Apixaban caused less bleeding than warfarin in patients with AF and CrCl 25 to 30 mL/min, with greater reductions in bleeding than in patients with CrCl > 30 mL/min
Sy J	2021	United States	Retrospective Cohort	351,407	Patients with AF and CKD	DOACs not specified	Warfarin	DOAC use associated with decreased risk of CV outcomes and lower risk of bleeding compared to warfarin for AF patients. DOACs maintained a superior safety profile across CKD stages
Yao X	2020	United States	Retrospective Cohort	34,569	Patients with AF across different stages of CKD	DOACs	Warfarin	Each DOAC (apixaban, dabigatran, rivaroxaban) consistently showed at least equivalent effectiveness and safety compared to warfarin across the range of kidney function
Fu C	2021	Taiwan	Retrospective Cohort	3,250	Patients with AF and CKD	Apixaban (standard dose: 10 mg/day; reduced dose: 2.5–5.0 mg/day)	Warfarin (INR goal range: 2–3)	Apixaban associated with reduced risk of stroke/SE and lower risk of major bleeding than warfarin in AF patients with eGFR < 30 ml/min/1.73 m^2^
Gurevitz C	2021	Israel	Prospective Cohort	2140	Patients with AF and renal impairment (eGFR < 60 ml/min/1.73 m^2^)	Apixaban (5 mg or 2.5 mg twice daily)	Warfarin (INR goal range: 2–3)	No significant difference in primary outcome and mortality between low-dose apixaban and warfarin. Apixaban appears to be a reasonable alternative to warfarin in renal impairment
Ahuja T	2021	United States	Retrospective Cohort	56	Patients with VTE and advanced CKD (Stage 4 or greater)	Apixaban or Rivaroxaban	Warfarin	Warfarin preferred in advanced CKD due to lack of renal elimination dependence. Apixaban found to be an acceptable, possibly safer, alternative to warfarin in advanced CKD
Shin J	2018	United States	Prospective Cohort	2244	Patients with AF and varying degrees of kidney function (eGFR ≥ 60, 30–59, < 30 ml/min/1.73 m^2^)	DOACs	Warfarin	Patients with eGFR < 60 ml/min/1.73 m^2^ on DOACs for AF had slightly higher bleeding risk than those on warfarin but similar benefits in ischemic stroke prevention
Coleman C	2019	Germany	Retrospective Cohort	6744	Patients with NVAF, stage 4 or 5 CKD, or undergoing hemodialysis	Rivaroxaban (standard dose: ≥ 20 mg/day; reduced dose: < 20 mg/day)	Warfarin (INR goal range: 2 to 3)	Rivaroxaban associated with 32% reduction in major bleeding compared to warfarin, with reductions in both intracranial and gastrointestinal bleeding in NVAF and CKD stages 4 or 5
Weir M	2020	United States	Retrospective Cohort	2317	Patients with NVAF and stage IV-V CKD	Rivaroxaban (standard dose: 15 mg/day for CrCl < 30 mL/min)	Warfarin (INR goal range: 2 to 3)	Comparable major bleeding risk between rivaroxaban and warfarin users. No significant difference in major bleeding rates between rivaroxaban and warfarin
Di Lullo L	2018	Italy	Retrospective Cohort	347	Patients with NVAF and moderate-to-advanced CKD (Stage 3b-4)	Rivaroxaban (15 mg once daily)	Warfarin (INR goal range: 2–3)	Rivaroxaban seems safe and effective in CKD stage 3b-4 patients. Future randomized controlled trials needed to definitively establish its role in CKD patients
Schafer J	2018	United States	Retrospective Cohort	604	Patients with NVAF or VTE and advanced CKD stages 4 and 5	Apixaban (2.5 mg or 5 mg twice daily)	Warfarin (INR goal range: 2–3)	Patients with advanced CKD taking apixaban had similar bleeding rates at 3 months compared to warfarin, but higher major bleeding rates with warfarin between 6 and 12 months
Makani A	2020	United States	Retrospective Cohort	20,208	Patients with NVAF and CKD	DOACs	Warfarin	DOACs safe and effective with lower mortality risk across all stages of renal impairment in patients with concomitant renal impairment and AF
Sarratt S	2017	United States	Retrospective Cohort	160	Patients with ESRD undergoing chronic hemodialysis	Apixaban (2.5 mg or 5 mg twice daily)	Warfarin (INR goal range: 2–3)	No difference in bleeding rates between apixaban and warfarin. Apixaban may be a cautious consideration in patients on hemodialysis
Chashkina M	2020	Russia	Randomized Controlled Trial	109	Patients with AF and stage 4 CKD or transient stable decline in GFR to 15–29 ml/min/1.73 m^2^	Rivaroxaban (15 mg daily)	Warfarin (INR goal range: 2–3)	Rivaroxaban has a favorable safety profile compared to warfarin in AF patients with advanced CKD
Chang S	2019	Taiwan	Retrospective Cohort	800	Patients with AF and renal dysfunction	DOACs	Warfarin	Both DOAC and warfarin use increased the risk of major bleeding
Laugesen E	2019	Denmark	Retrospective Cohort	1560	Patients with NVAF and CKD not receiving dialysis	DOACs	VKAs	Lower risk of major bleeding with DOACs in AF and CKD patients compared to VKA
Lee KH	2015	South Korea	Retrospective Cohort	1329	Patients with NVAF and CKD	DOACs	Warfarin	No difference in major bleeding risk between warfarin and DOACs, but significantly higher risk of major bleeding in warfarin users with renal dysfunction
Bonnemeier H	2019	Germany	Retrospective Cohort	8940	Patients with NVAF and renal impairment	Rivaroxaban (15 mg or 20 mg once daily)	Phenprocoumon (dose adjusted to maintain INR 2–3)	Lower incidence of intracranial hemorrhage with rivaroxaban compared to phenprocoumon in NVAF patients
Kee K	2023	Korea	Retrospective Cohort	1885	Patients with AF and renal dysfunction	DOACs	Warfarin	DOACs associated with a substantial reduction in risk of any ischemia and any bleeding compared to warfarin
Hsu C	2023	Taiwan	Retrospective Cohort	1011	Patients with AF and advanced kidney disease (AKD)	DOACs	Warfarin	DOACs may offer a potential safety advantage over warfarin, even in patients with a higher baseline risk

*CKD*: chronic kidney disease, *AF*: atrial fibrillation, *INR*: international normalized ratio, *DOACs*: direct oral anticoagulants, *VKA*: vitamin K antagonist, *HF*: heart failure, *VTE*: venous thromboembolism, *NVAF*: non-valvular atrial fibrillation, *eGFR*: estimated glomerular filtration rate, *CrCl*: creatinine clearance, *SE*: systemic embolism, *CV*: cardiovascular, *AKD*: advanced kidney disease, *ESRD*: end-stage renal disease

*Own authorship

### Meta-analysis results

A total of 19 studies were included in the following meta-analysis.

### Thromboembolism

**Fig. 3:**
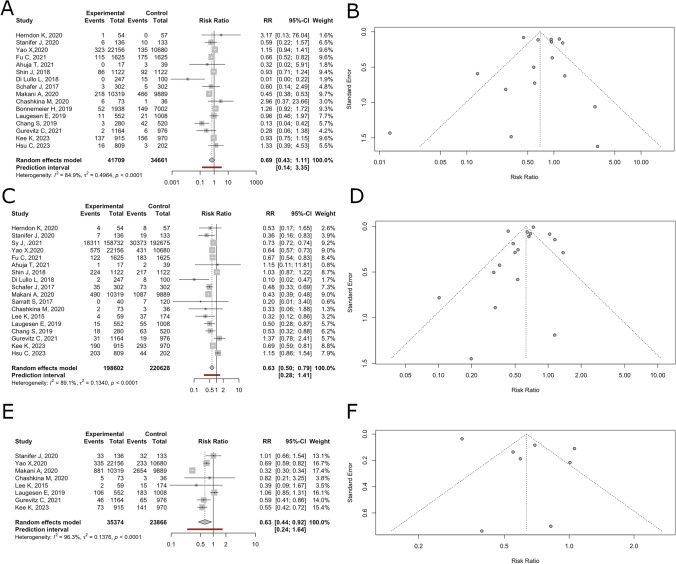
**A**. Forest plot detailing Relative Risk and 95% Confidence Intervals (CIs) of direct oral anticoagulants against vitamin K antagonists in thromboembolic events in patients with chronic kidney disease. **B**. Funnel plot of thromboembolic events in patients with chronic kidney disease. **C**. Forest plot detailing Relative Risk and 95% Confidence Intervals (CI) of direct oral anticoagulants against vitamin K antagonists in all-cause mortality events in patients with chronic kidney disease. **D**. Funnel plot of all-cause mortality events in patients with chronic kidney disease. **E**. Forest plot detailing Relative Risk and 95% Confidence Intervals (CIs) of direct oral anticoagulants against vitamin K antagonists in all-cause mortality events in patients with chronic kidney disease. **F**. Funnel plot of all-cause mortality events in patients with chronic kidney disease

In this meta-analysis assessing the effectiveness of direct oral anticoagulants compared to vitamin K antagonists in reducing thromboembolic events, data were pooled from 15 studies involving 76,370 participants. The general analysis using a random effects model indicated a non-significant reduction in thromboembolic events associated with direct oral anticoagulants with a RR = 0.69 (95% CI: 0.43–1.11, *p* = 0.11, *I*^2^ = 84.9%), with a wide prediction interval (0.14–3.28), suggesting variability in the effect sizes across different settings (Fig. 3A).

### Publication bias 

The funnel plot is asymmetric, suggesting possible publication bias; further assessment using Egger's test indicated no significant asymmetry (*t* = − 0.10, *p* = 0.92), suggesting minimal publication bias (Fig. 3B).

### Subgroup and sensitivity analysis

Subgroup analyses by risk of bias, stage, study design, and direct oral anticoagulant type showed no significant differences between subgroups. However, the follow-up and country subgroups revealed significant differences. Specifically, significant heterogeneity was observed concerning follow-up (*p* < 0.01), with different follow-up durations showing varying degrees of risk reduction. Significant differences were found with regard to country (*p* < 0.01), indicating variability in thromboembolic event risks across different countries. Notably, studies from the United States showed a non-significant risk reduction (RR = 0.74, 95% CI [0.47; 1.15], *I*^2^ = 90.0%), while studies from other countries displayed wide confidence intervals and varying effect sizes. A sensitivity analysis was performed, and the leave-one-out and influence analysis did not reveal any specific article influencing the overall result (Online Resources 10 and 11).

### Bleeding

In 18 studies comprising 419,230 participants, therapy with direct oral anticoagulants was associated with a statistically significant reduction in major bleeding events compared to vitamin K antagonists. The RR was 0.63 (95% CI: 0.50–0.79, *p* < 0.01), suggesting a robust effect across diverse clinical settings. However, significant heterogeneity was detected (*I* = 89.1%), indicating outcome variability across studies (Fig. 3C).

### Publication bias

Assessment of publication bias through funnel plot analysis and Egger’s regression indicated no significant asymmetry (*t* = − 1.23, *p* = 0.23), suggesting minimal publication bias affecting the results of the meta-analysis (Fig. 3D).

### Subgroup and sensitivity analysis

Subgroup analyses by risk of bias, study design, follow-up, and country revealed significant differences. Specifically, studies with some concerns of bias showed a significant risk reduction (RR = 0.32, 95% CI [0.25; 0.41], *I*^2^ = 0%) compared to low-risk studies (RR = 0.61, 95% CI [0.46; 0.80], *I*^2^ = 89.4%). Regarding study design, randomized controlled trials demonstrated a significant risk reduction (RR = 0.32, 95% CI [0.25; 0.41], *I*^2^ = 0%) compared to cohort studies (RR = 0.61, 95% CI [0.46; 0.80], *I*^2^ = 89.4%). Concerning the follow-up subgroup, significant differences were observed (*p* < 0.01), with various follow-up durations showing different degrees of risk reduction. Significant differences were found with regard to country (*p* = 0.01), indicating variability in major bleeding event risks across different countries. Notably, studies from the United States showed a significant risk reduction (RR = 0.58, 95% CI [0.42; 0.79], *I*^2^ = 93.2%), while other countries displayed wide confidence intervals and varying effect sizes. Sensitivity analyses, including leave-one-out and influence analyses, did not identify any specific study influencing the overall result significantly (Online Resources 12 and 13).

### All-cause mortality

The meta-analysis evaluated the impact of interventions on all-cause mortality and included eight studies comprising 59,240 participants. The pooled estimate using a random effects model demonstrated a significant reduction in mortality when using direct oral anticoagulants compared to vitamin K antagonists, with a RR of 0.63 (95% CI: 0.44–0.92, *p* = 0.02). The prediction interval ranged from 0.23 to 1.69, indicating considerable variability in the effect sizes across different settings. Heterogeneity was high (*I*^2^ = 96.3%), underscoring the variability among the included studies (Fig. 3E).

### Publication bias

The funnel plot shows asymmetry; however, the low number of studies included prevents a reliable assessment of publication bias (Fig. 3F).

### Subgroup and sensitivity analysis

Subgroup analyses by risk of bias, stage, study design, follow-up, and country revealed significant differences. Specifically, studies with a low risk of bias showed a significant risk reduction (RR = 0.54, 95% CI [0.31; 0.93], *I*^2^ = 97.5%) compared to those with some concerns (RR = 0.98, 95% CI [0.38; 2.49], *I*^2^ = 0%). Regarding stage, significant differences were observed (*p* < 0.01), with varying degrees of risk reduction across different stages. With regard to study design, cohort studies demonstrated a significant risk reduction (RR = 0.54, 95% CI [0.31; 0.93, 12 = 97.5%) compared to randomized controlled trials (RR = 0.98, 95% CI [0.38;2.49], *I*^2^ = 0%), while significant differences were found for follow-up (*p* < 0.01), with different follow-up durations showing varying effect sizes. Concerning country, significant differences were also found (*p* < 0.01), indicating variability in all-cause mortality risks across different countries. Notably, studies from the United States showed a non-significant risk reduction (RR = 0.54, 95% CI [0.09; 3.19], *I*^2^ = 98.4%), while other countries displayed wide confidence intervals and varying effect sizes. The sensitivity analysis, including leave-one-out and influence, identified specific studies, like the one by Makani et al., that significantly affected heterogeneity and overall outcomes [41]. This highlights the need to evaluate influential studies carefully to ensure robust findings. The exclusion of one study (Makani et al.) due to influential effects significantly altered the pooled effect size RR = 0.74 (95% CI: 0.56–0.97), highlighting the sensitivity of the meta-analysis results to individual studies. This adjustment also substantially reduced heterogeneity (*I*^2^ = 70% (Online Resources 14 and 15)).

## Discussion

Patients with CKD and atrial fibrillation are at risk of presenting both thromboembolic and bleeding events [53–55]. Since the introduction of direct oral anticoagulants in the early 2010s, a new alternative for anticoagulation, with ergonomic administration and no need for laboratory coagulation quantification became available [56]. However, when trying to prevent thromboembolism, patients with advanced CKD and kidney failure pose another challenge; it is believed that the pharmacokinetics of direct oral anticoagulants outweigh their benefits in this population [57]. For decades, vitamin K antagonists have been the mainstay therapy in patients requiring thromboembolism prophylaxis for atrial fibrillation in the setting of chronic kidney disease or ESKD. Data backing up this practice are limited, as randomized clinical trials have excluded patients with CKD stages 3, 4, and ESKD [57]. This meta-analysis yielded a RR = 0.69 (95% CI: 0.43–1.11, *p* = 0.11) for thromboembolic events in patients taking direct oral anticoagulants compared to those on vitamin K antagonists. Although this suggests a trend towards fewer thromboembolic events among patients receiving direct oral anticoagulants, the results were not statistically significant. The heterogeneity was substantial (*I*^2^ = 84.9%), with a highly significant test for heterogeneity (*p* < 0.01), indicating considerable variability in the effect sizes across the different studies and elucidating the importance of continuing to perform clinical trials that target CKD and ESKD patients, under similar circumstances. Subgroup analysis revealed that country and follow-up duration are sources of heterogeneity; this finding emphasizes the potential influence of regional clinical practices, patient demographics, healthcare systems, and the importance of longer follow-up to provide a more accurate assessment of the effectiveness of anticoagulation therapies. A recent meta-analysis pointed out the superiority of direct oral anticoagulants over vitamin K antagonists in patients with atrial fibrillation [78]. Despite this evidence, the authors did not focus on CKD patients; our results suggest that direct oral anticoagulants in our specific population could provide benefits, but there is still a need to obtain high quality studies to further evaluate these options and reduce the heterogeneity [58]. It is important to point out that the risk of thromboembolism in CKD patients increases as kidney function decreases, and that concomitant atrial fibrillation increases with the progression of CKD; for this reason, it is important to measure and evaluate the effectiveness of the direct oral anticoagulants [59–62]. It is important to mention that preventing adverse events would improve the quality of life and economic medical burden [62–64]. Nevertheless, previous studies in several populations at high risk for thromboembolism, such as cancer patients, have shown the efficacy and safety of direct oral anticoagulants. Also, some meta-analyses have shown efficacy and safety in the pediatric population [65–67].

In our initial analysis, bleeding events among patients on direct oral anticoagulants, compared with vitamin K antagonists, showed a RR = 0.63 (95% CI: 0.50–0.79, *p* < 0.01), indicating a non-statistically significant reduced risk compared to vitamin K antagonists. The implications for future treatments are significant, suggesting a potential advantage in favor of direct oral anticoagulants over vitamin K antagonists regarding bleeding risk [68, 69]. Future investigations should address the sources of heterogeneity observed in this meta-analysis, such as the study design showing a marked reduction in randomized controlled trials, follow-up, country, and risk of bias. This suggests that large-scale prospective studies with standardized protocols and diverse patient populations are essential to validate and extend our findings beyond our meta-analysis findings. Several recent meta-analyses in different populations have underscored the superiority, efficacy, and safety of direct oral anticoagulants versus vitamin K antagonists in bleeding among different populations, aligning with our results in the CKD population [70–72]. Acknowledging the wide range of direct oral anticoagulants evaluated in our analysis and the prevalence of warfarin as the primary vitamin K antagonist in the studies reviewed, it becomes imperative to consider the nuances specific to each medication and their potential impact on bleeding risk. The last of our analyses examines the association between direct oral anticoagulants and vitamin K antagonists and all-cause mortality with a RR = 0.63 (95% CI: 0.44–0.92, *p* = 0.02), indicating a significant advantage of direct oral anticoagulants, but with high heterogeneity. After removing the articles that contributed most to the heterogeneity, the overall effect remained statistically significant. In a previous report by Harrington et al. involving 71,683 patients with a mean creatinine clearance of 75.5 ± 0.5 mL/min, it was shown that the incidence of death increased significantly with worsening kidney function, which is consistent with our subgroup analysis by stage [73]. There was a discernible trend towards increasing benefit from standard-dose direct oral anticoagulants as kidney function declined, with hazard ratios decreasing by 2.1% (ranging from -0.3% to 4.4%) for every 10 mL/min decrease in creatinine clearance. Although the trend did not reach statistical significance (*p* = 0.08), the findings suggest a potential advantage of standard-dose direct oral anticoagulants over warfarin in patients with impaired kidney function, which aligns with our findings [73]. In another analysis by Zeng et al., encompassing four studies and 835,520 patients, direct oral anticoagulants were compared with warfarin in atrial fibrillation patients with frailty. Direct oral anticoagulant therapy was associated with reduced risks of stroke or systemic embolism, ischemic stroke, hemorrhagic stroke, and all-cause death. These findings align with our results in our specific population, suggesting an evident superiority of direct oral anticoagulants over vitamin K antagonists [74]. Some other meta-analyses involving different populations, such as patients with hip trauma and intracranial hemorrhage, have found direct oral anticoagulants to be useful in reducing mortality, showing the efficacy and safety of this medication among different populations[75, 76]. These analyses suggest that direct oral anticoagulant therapy exhibits benefits in terms of reduced mortality and improved safety outcomes compared to warfarin in CKD patients.

This study presents several limitations that should be acknowledged. First, the included studies exhibit significant heterogeneity in design, ranging from retrospective and prospective cohorts to randomized controlled trials. This variability has been shown to affect the consistency and robustness of the findings in the subgroup analysis and should be interpreted cautiously. Additionally, the patient populations studied vary widely, encompassing different stages of CKD, age groups, and comorbid conditions. This may limit the generalizability of the findings, as the absence of significant differences in the subgroup analysis of CKD stages may be attributed to the limited number of studies for some subgroups. There are also inconsistencies in the specific interventions and comparators across studies. Some studies focused on different doses or types of direct oral anticoagulants, while others did not clearly specify the type of anticoagulant used. These differences can complicate direct comparisons and synthesis of the findings. Furthermore, many studies are observational, which inherently carries a higher risk of bias and confounding factors than randomized controlled trials. These limitations underscore the need for cautious interpretation of the results and highlight the importance of conducting further high-quality, large-scale randomized trials to confirm these findings and provide more definitive guidance for clinical practice.

## Conclusions

Our findings support the choice of direct oral anticoagulants over vitamin K antagonists in certain CKD contexts. However, given the complexities of CKD, anticoagulation therapy decisions should be tailored to the individual patient, informed by the latest and most comprehensive evidence. Future research is needed to enhance our understanding and improve patient outcomes in this challenging clinical area.

## Supplementary Information

Below is the link to the electronic supplementary material.

Supplementary file1 (DOCX 55 KB)

## Data Availability

Data supporting this study's findings are available from the corresponding author upon reasonable request.
