# Supplementary material for: Efficacy and safety of novel anticoagulant therapies in patients with chronic kidney disease—a systematic review and meta-analysis
Source: J Nephrol. 2024 Nov 29;38(1):111–26. doi: 10.1007/s40620-024-02130-3 (PMC11903568; doi:10.1007/s40620-024-02130-3)
Supplement: Supplementary file 1 — Supplementary file1 (DOCX 55 KB) [file 40620_2024_2130_MOESM1_ESM.docx]

1

**Efficacy and safety of novel anticoagulant therapies in patients with chronic kidney disease- Systematic review and meta-analysis**

**Journal of Nephrology**

**Authors:**

Ernesto Calderon Martinez^1^, Camila Sanchez Cruz^1^, Edna Y. Diarte Acosta^2^, Daniel Alejandro Aguirre Cano^3^,

Ana Maria Espinosa^4^, Diana Othón Martínez ^5^, Flor Furman ^6^, Sebastian Obando Vera^7^

^1^Universidad Nacional Autónoma de México, Ciudad de México, México.

2Universidad Autónoma de Sinaloa, Sinaloa, México.

^3^Universidad de Monterrey, Monterrey, México.

^4^Univesidad Nacional de Loja, Loja, Ecuador.

^5^University of Texas RGV, Texas, USA.

^6^Universidad de Buenos Aires, Buenos Aires, Argentina.

^7^Universidad Catolica de Santa Maria, Arequipa, Perú.

**Corresponding author:** Ernesto Calderon Martinez, ernestocalderon.mtz@gmail.com, ORCID ID: 00000003-2237-9536, Academic, Department of Biomedical Informatics, Universidad Nacional Autonoma de Mexico

**Online Resource 1** PubMed

| **Search** |  | **Result** |
| --- | --- | --- |
| (("Anticoagulants"[Mesh] OR  "Anticoagulants"[Title/Abstract] OR  "Anticoagulant Therapy"[Title/Abstract] OR  ‘’Anticoagulan*’’[Title/Abstract] OR  "Antithrombins"[Title/Abstract] OR "Thrombin  Inhibitors"[Title/Abstract] OR "Factor Xa Inhibitors"[Title/Abstract] OR ‘’Vitamin K  Inhibi*’’[Title/Abstract] OR  "dabigatran"[Title/Abstract] OR  "apixaban"[Title/Abstract] OR  "rivaroxaban"[Title/Abstract] OR "warfarin"  [Title/Abstract]) AND ("Kidney Diseases"[Mesh] OR ‘’Kidney Disease*’’[Title/Abstract] OR  "Chronic Kidney Disease"[Title/Abstract] OR  ‘’CKD’’[Title/Abstract] OR "Renal  Insufficiency"[Title/Abstract] OR "Renal  Failure"[Title/Abstract] OR "End-Stage Kidney  Disease"[Title/Abstract] OR "End-Stage renal Disease"[Title/Abstract]) OR "Kidney disease"[Title/Abstract] OR "chronic renal  insufficiency"[Title/Abstract])AND (‘’ trials, randomized clinical’’[Mesh] OR "Randomized  Control Trials"[Title/Abstract] OR "Clinical  Trials""[Title/Abstract] OR ‘’RCT’’[Title/Abstract] OR ‘’Casecontrol’’[Title/Abstract] OR ‘’Cohort’’[Title/Abstract])) | **21,053** |  |

***PubMed: Extracted January 10, 2024**

**Online Resource 2** EMBASE

| **Item** | **Search** | **Results** |
| --- | --- | --- |
| 1 | anticoagulants | 61,623 |
| 2 | anticoagulants':ab,jt | 48,449 |
| 3 | anticoagulant therapy':ab,jt | 66,668 |
| 4 | anticoagulan*':ab,jt | 114,690 |
| 5 | antithrombins':ab,jt | 235 |
| 6 | thrombin inhibitors':ab,jt | 10,568 |
| 7 | factor xa inhibitors':ab,jt | 3,922 |
| 8 | vitamin k inhibi*':ab,jt | 14,965 |
| 9 | dabigatran':ab,jt | 10,908 |
| 10 | apixaban':ab,jt | 10,774 |
| 11 | rivaroxaban':ab,jt | 14,568 |
| 12 | warfarin':ab,jt | 43,420 |
| 13 | kidney diseases' | 226,350 |
| 14 | kidney diseas*':ab,jt | 552,970 |
| 15 | chronic kidney disease':ab,jt | 205,215 |
| 16 | ckd':ab,jt | 86,123 |
| 17 | renal insufficiency':ab,jt | 39,186 |
| 18 | renal failure':ab,jt | 213,067 |
| 19 | end-stage kidney disease':ab,jt | 73,166 |
| 20 | end-stage renal disease':ab,jt | 82,489 |
| 21 | kidney disease':ab,jt | 477,620 |
| 22 | chronic renal insufficiency':ab,jt | 14,151 |
| 23 | trials, randomized clinical' | 332,083 |
| 24 | randomized control trials':ab,jt | 96,363 |
| 25 | clinical trials':ab,jt | 829,746 |
| 26 | rct':ab,jt | 54,827 |
| 27 | case-control':ab,jt | 191,027 |
| 28 | cohort':ab,jt | 1,342,263 |
| 29 | #1 OR #2 OR #3 OR #4 OR #5 OR #6 OR #7 OR #8 OR #9 OR #10 OR #11 OR  #12 | 199,111 |
| 30 | #13 OR #14 OR #15 OR #16 OR #17 OR #18 OR #19 OR #20 OR #21 OR #22 | 769,298 |
| 31 | #23 OR #24 OR #25 OR #26 OR #27 OR #28 | 2,332,573 |
| 32 | #29 AND #30 AND #31 | 2550 |

* **EMBASE: Extracted January 10, 2024**

**Online Resource 3.** Cochrane library

| **Item** | **Search** | **Results** |
| --- | --- | --- |
| 1 | MeSH descriptor: [Anticoagulants] explode all trees. | 7121 |
| 2 | (anticoagulants):ti,ab,kw | 8739 |
| 3 | (anticoagulant therapy):ti,ab,kw | 5582 |
| 4 | (anticoagulan*):ti,ab,kw | 13627 |
| 5 | (antithrombins):ti,ab,kw | 531 |
| 6 | (thrombin inhibitors):ti,ab,kw | 893 |
| 7 | (vitamin k inhibitors):ti,ab,kw | 504 |
| 8 | (factor Xa inhibitors):ti,ab,kw | 1103 |
| 9 | (dabigatran):ti,ab,kw | 1185 |
| 10 | (apixaban):ti,ab,kw | 1160 |
| 11 | ("rivaroxaban"):ti,ab,kw | 2353 |
| 12 | ("Warfarin"):ti,ab,kw | 5482 |
| 13 | #1 OR #2 OR #3 OR #4 OR #5 OR #6 OR #7 OR #8 OR #9 OR #10 OR  #11 OR #12 | 18274 |
| 14 | MeSH descriptor: [Renal Insufficiency, Chronic] explode all trees | 9641 |
| 15 | (kidney disease):ti,ab,kw | 32780 |
| 16 | ("end stage renal disease"):ti,ab,kw | 6628 |
| 17 | (renal failure):ti,ab,kw | 23230 |
| 18 | (kidney diseas*):ti,ab,kw | 36856 |
| 19 | (CKD):ti,ab,kw | 8123 |
| 20 | (chronic kidney disease):ti,ab,kw | 16573 |
| 21 | ("chronic renal insufficiency"):ti,ab,kw | 5456 |
| 22 | #14 OR #15 #16 OR #17 OR #18 OR #19 OR #20 OR #21 | 51894 |
| 23 | MeSH descriptor: [Randomized Controlled Trial] explode all trees. | 37 |
| 24 | (trials, randomized clinical):ti,ab,kw | 179350 |
| 25 | (RCT):ti,ab,kw | 40346 |
| 26 | ("randomized control trial"):ti,ab,kw | 321897 |
| 27 | ("case control studies"):ti,ab,kw | 18548 |
| 28 | (cohort studies):ti,ab,kw | 35617 |
| 29 | #23 OR #24 OR #25 OR #26 OR #27 OR #28 | 495077 |
| 30 | #13 AND #23 AND #29 | 368 |

***Cochrane library: Extracted January 10, 2024**

**Online Resource 4:** Web of Science

| **Item** | **Search** | **Results** |
| --- | --- | --- |
| 1 | KP=(Anticoagulants) | 18618 |
| 2 | AB=(anticoagulants) | 55478 |
| 3 | TI=(anticoagulants) | 29475 |
| 4 | TI= (anticoagulant therapy) | 4756 |
| 5 | AB= (anticoagulant therapy) | 19287 |
| 6 | AB=(anticoagulan*) | 55517 |
| 7 | TI=(anticoagulan*) | 29568 |
| 8 | TI=(antithrombins) | 57 |
| 9 | AB=(antithrombins) | 113 |
| 10 | AB= (thrombin inhibitors) | 12975 |
| 11 | TI= (thrombin inhibitors) | 3585 |
| 12 | TI= (vitamin k inhibitors) | 100 |
| 13 | AB= (vitamin k inhibitors) | 2176 |
| 14 | AB= (factor Xa inhibitors) | 4161 |
| 15 | TI= (factor Xa inhibitors) | 1886 |
| 16 | AB=(dabigatran) | 4877 |
| 17 | TI=(dabigatran) | 3466 |
| 18 | TI=(apixaban) | 2296 |
| 19 | AB=(apixaban) | 4469 |
| 20 | AB=(rivaroxaban) | 6528 |
| 21 | TI=(rivaroxaban) | 4477 |
| 22 | TI=(warfarin) | 14293 |
| 23 | AB=(warfarin) | 21011 |
| 24 | TI=(warfarin) | 14293 |
| 25 | #24 OR #23 OR #22 OR #21 OR #20 OR #19 OR #18 OR #17 OR  #16 OR #15 OR #14 OR #13 OR #12 OR #11 OR #10 OR #9 OR #8 OR #7 OR #6 OR #5 OR #4 OR #3 OR #2 OR #1 | 120934 |
| 26 | KP= (Chronic Renal Insufficiency) | 1034 |
| 27 | TI= (chronic renal insufficiency) | 2131 |
| 28 | AB= (chronic renal insufficiency) | 5322 |
| 29 | TI= (End-Stage Kidney Disease) | 2716 |
| 30 | AB= (End-Stage Kidney Disease) | 23040 |
| 31 | AB= (Renal Insufficiency) | 15924 |
| 32 | TI= (Renal Insufficiency) | 6730 |
| 33 | TI= (Chronic Kidney Disease) | 39180 |
| 34 | AB= (Chronic Kidney Disease) | 80463 |
| 35 | AB= (Kidney Diseas*) | 171686 |
| 36 | TI= (Kidney Diseas*) | 64676 |
| 37 | KP= (Kidney Diseases) | 55398 |
| 38 | TI=(CKD) | 9363 |
| 39 | AB=(CKD) | 43176 |
| 40 | AB= (kidney diseas*) | 171686 |
| 41 | TI= (kidney diseas*) | 64676 |
| 42 | TI= (renal failure) | 40070 |
| 43 | AB= (renal failure) | 89730 |
| 44 | AB= (end stage renal disease) | 39257 |
| 45 | TI= (end stage renal disease) | 12329 |
| 46 | TI= (kidney disease) | 64420 |
| 47 | AB= (kidney disease) | 170936 |
| 48 | #26 OR #27 OR #28 OR #29 OR #30 OR #31 OR #32 OR #33 OR #34 OR #35 OR #36 OR #37 OR #38 OR #39 OR #40 OR #41 OR  #42 OR #43 OR #44 OR #45 OR #46 OR #47 | 368923 |
| 49 | #48 AND #25 | 3730 |

***Web of Science: Extracted January 10, 2024 Online Resource 5** Biblioteca Virtual en Saludà Medline, IBECS, WPRIM, LILACS

| **Search** |  | **Results** |
| --- | --- | --- |
| (mj:(Anticoagulants) OR (Anticoagulant  Therapy) OR (Anticoagulan*) OR  (Antithrombins) OR (Thrombin Inhibitors) OR  (Factor Xa Inhibitors) OR (Vitamin K Inhibi*)  OR (dabigatran) OR (apixaban) OR  (rivaroxaban) OR (warfarin) AND  (mj:(Kidney Diseases)) OR (Kidney Diseas*)  OR (Chronic Kidney Disease) OR (CKD) OR (Renal Insufficiency) OR (Renal Failure) OR (End-Stage Kidney Disease) OR (End-Stage renal Disease) OR (Kidney disease) OR  (chronic renal insufficiency) AND (mj:(trials, randomized clinical)) OR (Randomized Control  Trials) OR (Clinical Trials) OR (RCT) OR (Case-control) | **347** |  |

***Own authorship**

**Online Resource 6** Clinicaltrials.gov

| **Search** |  | **Result** |
| --- | --- | --- |
| CONDITION: Chronic Kidney disease OR  ''Kidney Diseas*'' OR ''Chronic Kidney  Disease'' OR ''CKD'' OR ''Renal Insufficiency''  OR ''Renal Failure'' OR ''End-Stage Kidney  Disease'' OR ''End-Stage renal Disease'' OR  ''Kidney disease'' OR ''chronic renal insufficiency''  STUDY TYPE: Interventional/Clinical Trial  INTERVENTION: ‘’Anticoagulants’’ OR  ‘’Anticoagulant Therapy’’ OR ‘’Anticoagulan*’’  OR ‘’Antithrombins’’ OR ‘’Thrombin Inhibitors’’  OR ‘’Factor Xa Inhibitors’’ OR ‘’Vitamin K  Inhibi*’’ OR ‘’dabigatran’’ OR ‘’apixaban’’ OR  ‘’rivaroxaban’’ OR ‘’warfarin’’ | 191 |  |

***Own authorship**

**Online Resource 7** World Health Organization International Clinical Trials Registry Platform (ICTRP) à 1903

| **Search** |  | **Results** |
| --- | --- | --- |
| Title: (Anticoagulants) OR (Anticoagulant) OR  (Anticoagulant Therapy) OR (Anticoagulan*)  OR (Antithrombins) OR (Thrombin Inhibitors)  OR (Factor Xa Inhibitors) OR (Vitamin K  Inhibi*) OR (dabigatran) OR (apixaban) OR  (rivaroxaban) OR (warfarin) OR (Kidney  Diseases) OR (Kidney Diseas*) OR (Chronic | 2184 |  |
| Kidney Disease) OR (CKD) OR (Renal  Insufficiency) OR (Renal Failure) OR (EndStage Kidney Disease) OR (End-Stage renal Disease) OR (Kidney disease) OR (chronic renal insufficiency)  Disease: (Kidney Diseases) OR (Kidney  Diseas*) OR (Chronic Kidney Disease) OR  (CKD) OR (Renal Insufficiency) OR (Renal  Failure) OR (End-Stage Kidney Disease) OR (End-Stage renal Disease) OR (Kidney disease) OR (chronic renal insufficiency)  Intervention: (Anticoagulants) OR  (Anticoagulant) OR (Anticoagulant Therapy)  OR (Anticoagulan*) OR (Antithrombins) OR  (Thrombin Inhibitors) OR (Factor Xa Inhibitors)  OR (Vitamin K Inhibi*) OR (dabigatran) OR  (apixaban) OR (rivaroxaban) OR (warfarin) |  | |

***Own authorship**

**Online Resource 8** Inclusion / Exclusion criteria

| **Inclusion** | **Exclusion** |
| --- | --- |
| Studies in patients with chronic kidney disease | Population-based cross-sectional studies |
| Studies in patients with chronic prerenal – postrenal disease | Studies that are not related to safety and efficacy of treatment. |
| Studies that report efficacy and safety of DOACs | Studies that report on animal models |
| Studies that include human subjects | Studies that do not report original data. |
| Studies that include patients >18 years of any gender | Systematic reviews |
| Studies that are written in English or Spanish | Studies that are not available in full text or cannot be obtained through interlibrary loan. |
| Prospective or retrospective cohort | Studies of case series |
| Case-control studies | Studies that include patients <18 years of any gender |
| Studies between 2013-2023 | Studies that are written in other language that isn’t  English or Spanish |
| Randomized clinical trials. | Studies that report patients with hemophilia with low levels of either factor VIII or factor XI |
| Meta-analysis | Dissertations |
| Risk factors for major bleed based on stratification risk score (CHA2DS2-VASc) | Self-reported diagnosis of CKD |
| Unfractionated heparin/warfarin inclusion as contrast studies | Book chapters |
|  | Protocol articles |
|  | Reviews |
|  | News articles |
|  | Conference abstracts |
|  | Letters to the editor |
|  | Editorials |
|  | Comment publications |
|  | Studies with no description of their operationalization |
|  | Incomplete reporting |
|  | Studies based on experimental anticoagulants or in any investigational phase are not approved by the FDA. |
|  | The use of anticoagulants for other medical conditions, such as AF. |
|  | Case reports |
|  | Guidelines |

*Own authorship

**Online Resource 9** Highlights Inclusion / Exclusion criteria

| **Inclusion:** | **Exclusion:** |
| --- | --- |
| CKD | Animals |
| Chronic kidney disease | Animal |
| Chronic kidney failure | Animal models |
| Kidney failure | Model animals |
| RR | Models |
| OR | Animals models |
| Odds ratio | Murine |
| Odds ratios | Murines |
| Relative risk | Pediatric |
| Risk Ratio | Kid |
| Odds | Kids |
| Odd ratio | Children |
| Safety | <18 years |
| Efficacy | Experimental phase |
| Sex | Investigational phase |

| Female | Phase 1 |
| --- | --- |
| Male | Phase 0 |
| Age | AF |
| >18 years | Hemophilia |
| CHADS2 score | Factor VII |
| CHA2DS2-VASc score | Factor XI |
| Major bleeding | Studies before 2013 |
| Minor bleedings | Review |
| Bleeding | Conference |
| bleedings | Conferences |
| Stroke | Abstract |
| Brain Attack | Comment |
| Ruptured artery | Comments |
| Embolism | Letter |
| Blocked artery | Letters |
| Mortality | Editorial |
| Death rate |  |
| All-cause |  |
| All-cause mortality |  |
| Allcause |  |
| Heparin/Warfarin |  |
| Unfractionated Heparin/Warfarin |  |
| UFH |  |
| Glycosaminoglycan |  |
| Period of time from 2013-2023 |  |
| DOAC |  |
| HAS-BLED score |  |
| Human subjects |  |
| Human |  |
| Randomized Clinical Trials |  |
| Novel Anticoagulants |  |
| Novel |  |
| Anticoagulants |  |
| New Therapies |  |
| Therapies |  |

*Own authorship

**Online Resource 10** Subgroup analysis for Thromboembolic events in patients with Chronic kidney disease.

| **Variable** | **Subgroup** | **k** | **OR** | **95%-CI** | **I² (%)** | **p-value (Between Groups)** |
| --- | --- | --- | --- | --- | --- | --- |
| **Risk of Bias** | Low | 14 | 0.64 | [0.37; 1.12] | 86.7 | 0.59 |
|  | Some Concerns | 2 | 1.02 | [0.00; 29997.07] | 48.9 |  |
| **Stage** | >4 | 7 | 0.63 | [0.22; 1.79] | 49.8 | 0.29 |
|  | All | 4 | 0.96 | [0.58; 1.56] | 82.8 |  |
|  | >3 | 5 | 0.38 | [0.06; 2.48] | 86.0 |  |
| **Study Design** | Cohort | 14 | 0.64 | [0.37; 1.12] | 86.7 | 0.59 |
|  | RCT | 2 | 1.02 | [0.00; 29997.07] | 48.9 |  |
| **DOAC** | Apixaban | 5 | 0.62 | [0.49; 0.79] | 0.0 | 0.96 |
|  | DOACs | 7 | 0.67 | [0.32; 1.38] | 91.3 |  |
|  | Rivaroxaban | 4 | 0.56 | [0.01; 21.74] | 77.4 |  |
| **Follow-up** | Not specified | 5 | 0.79 | [0.21; 3.00] | 73.9 | < 0.01 |
|  | 2 | 3 | 0.81 | [0.31; 2.12] | 86.3 |  |
|  | 0.25 | 1 | 0.30 | [0.01; 6.09] | -- |  |
|  | 1.2 | 1 | 0.93 | [0.68; 1.26] | -- |  |
|  | 1.3 | 1 | 0.01 | [0.00; 0.19] | -- |  |
|  | 1 | 3 | 0.74 | [0.20; 2.77] | 0.0 |  |
|  | 3.4 | 1 | 0.44 | [0.37; 0.51] | -- |  |
|  | 1.5 | 1 | 3.13 | [0.36; 27.07] | -- |  |
| **Country** | United States | 7 | 0.74 | [0.48; 1.16] | 90.0 | 0.01 |
|  | Taiwan | 3 | 0.48 | [0.03; 8.68] | 76.9 |  |
|  | Italy | 1 | 0.01 | [0.00; 0.19] | -- |  |
|  | Russia | 1 | 3.13 | [0.36; 27.07] | -- |  |
|  | Germany | 1 | 1.27 | [0.92; 1.75] | -- |  |
|  | Denmark | 1 | 0.96 | [0.46; 2.00] | -- |  |
|  | Israel | 1 | 0.28 | [0.06; 1.38] | -- |  |
|  | Korea | 1 | 0.92 | [0.72; 1.18] | -- |  |

**Online Resource 11** Leave-one-out analysis for Thromboembolic events in patients with Chronic kidney disease.

| **Author** | **Effect** | **95% CI: Lower** | **95% CI: Upper** | **I^2** | **RStudent** | **DFFITS** | **Cook's D** | **Cov. R** | **Tau² (del)** | **QE (del)** | **Hat** | **Weight** | **Is Influential** |
| --- | --- | --- | --- | --- | --- | --- | --- | --- | --- | --- | --- | --- | --- |
| Omitting Herndon K, 2020 | 0.68 | 0.42 | 1.10 | 0.86 | 0.86 | 0.12 | 0.02 | 1.05 | 0.52 | 98.34 | 0.02 | 1.56 | no |
| Omitting Stanifer J, 2020 | 0.69 | 0.41 | 1.17 | 0.86 | -0.18 | -0.00 | 0.00 | 1.21 | 0.58 | 98.91 | 0.07 | 6.52 | no |
| Omitting Yao X, 2020 | 0.65 | 0.39 | 1.09 | 0.82 | 0.73 | 0.26 | 0.07 | 1.17 | 0.54 | 76.72 | 0.10 | 9.62 | no |
| Omitting Fu C, 2021 | 0.69 | 0.41 | 1.17 | 0.86 | -0.06 | 0.02 | 0.00 | 1.25 | 0.58 | 97.70 | 0.10 | 9.57 | no |
| Omitting Ahuja T, 2021 | 0.70 | 0.42 | 1.16 | 0.86 | -0.46 | -0.03 | 0.00 | 1.13 | 0.56 | 98.82 | 0.02 | 1.81 | no |
| Omitting Shin J, 2018 | 0.67 | 0.40 | 1.13 | 0.85 | 0.42 | 0.17 | 0.03 | 1.22 | 0.57 | 96.44 | 0.09 | 9.43 | no |
| Omitting Di Lullo L, 2018 | 0.77 | 0.55 | 1.07 | 0.85 | -2.70 | -0.50 | 0.22 | 0.49 | 0.19 | 91.16 | 0.02 | 1.91 | no |
| Omitting Schafer J, 2017 | 0.69 | 0.41 | 1.16 | 0.86 | -0.13 | 0.01 | 0.00 | 1.19 | 0.58 | 99.05 | 0.05 | 4.77 | no |
| Omitting Makani A, 2020 | 0.72 | 0.43 | 1.21 | 0.67 | -0.61 | -0.17 | 0.03 | 1.20 | 0.55 | 42.89 | 0.10 | 9.70 | no |
| Omitting Chashkina M, 2020 | 0.67 | 0.42 | 1.07 | 0.86 | 1.16 | 0.18 | 0.03 | 0.99 | 0.47 | 97.45 | 0.03 | 3.01 | no |
| Omitting Bonnemeier H, 2019 | 0.65 | 0.39 | 1.08 | 0.84 | 0.85 | 0.28 | 0.08 | 1.14 | 0.52 | 87.37 | 0.09 | 9.35 | no |
| Omitting Laugesen E, 2019 | 0.67 | 0.40 | 1.13 | 0.86 | 0.41 | 0.16 | 0.03 | 1.20 | 0.57 | 98.68 | 0.08 | 7.71 | no |
| Omitting Chang S, 2019 | 0.79 | 0.53 | 1.16 | 0.85 | -2.19 | -0.66 | 0.33 | 0.67 | 0.28 | 90.61 | 0.06 | 5.75 | no |
| Omitting Gurevitz C, 2021 | 0.72 | 0.44 | 1.18 | 0.86 | -0.85 | -0.17 | 0.03 | 1.08 | 0.52 | 97.68 | 0.04 | 4.20 | no |
| Omitting Kee K, 2023 | 0.67 | 0.39 | 1.13 | 0.85 | 0.42 | 0.17 | 0.03 | 1.23 | 0.57 | 94.08 | 0.10 | 9.60 | no |
| Omitting Hsu C, 2023 | 0.66 | 0.40 | 1.10 | 0.86 | 0.70 | 0.20 | 0.04 | 1.13 | 0.54 | 98.27 | 0.06 | 5.50 | no |

**Online Resource 12** Subgroup analysis for Bleeding events in patients with Chronic kidney disease.

| **Variable** | **Subgroup** | **k** | **OR** | **95%-CI** | **I² (%)** | **p-value (Between Groups)** |
| --- | --- | --- | --- | --- | --- | --- |
| **Risk of Bias** | Low | 16 | 0.61 | [0.46; 0.81] | 89.4 | < 0.01 |
|  | Some Concerns | 2 | 0.32 | [0.25; 0.41] | 0.0 |  |
| **Stage** | >4 | 7 | 0.57 | [0.35; 0.94] | 68.4 | 0.78 |
|  | >3 | 7 | 0.55 | [0.27; 1.13] | 95.1 |  |
|  | All | 3 | 0.63 | [0.59; 0.67] | 0.0 |  |
|  | >5 | 1 | 0.19 | [0.01; 3.35] | -- |  |
| **Study Design** | Cohort | 16 | 0.61 | [0.46; 0.81] | 89.4 | < 0.01 |
|  | RCT | 2 | 0.32 | [0.25; 0.41] | 0.0 |  |
| **DOAC** | Apixaban | 6 | 0.58 | [0.32; 1.05] | 63.0 | 0.49 |
|  | DOACs | 9 | 0.64 | [0.46; 0.90] | 93.5 |  |
|  | Rivaroxaban | 3 | 0.33 | [0.03; 3.45] | 65.5 |  |
| **Follow-up** | Not specified | 6 | 0.61 | [0.35; 1.07] | 67.5 | < 0.01 |
|  | 2 | 3 | 0.62 | [0.44; 0.88] | 3.7 |  |
|  | 0.28 | 1 | 0.70 | [0.68; 0.71] | -- |  |
|  | 0.25 | 1 | 1.16 | [0.10; 13.68] | -- |  |
|  | 1.2 | 1 | 1.04 | [0.84; 1.28] | -- |  |
|  | 1.3 | 1 | 0.09 | [0.02; 0.45] | -- |  |
|  | 1 | 3 | 0.64 | [0.13; 3.25] | 82.3 |  |
|  | 3.4 | 1 | 0.40 | [0.36; 0.45] | -- |  |
|  | 1.5 | 1 | 0.31 | [0.05; 1.94] | -- |  |
| **Country** | United States | 9 | 0.58 | [0.43; 0.79] | 93.2 | 0.02 |
|  | Taiwan | 3 | 0.74 | [0.24; 2.23] | 80.1 |  |
|  | Italy | 1 | 0.09 | [0.02; 0.45] | -- |  |
|  | Russia | 1 | 0.31 | [0.05; 1.94] | -- |  |
|  | South Korea | 1 | 0.27 | [0.09; 0.79] | -- |  |
|  | Denmark | 1 | 0.48 | [0.27; 0.87] | -- |  |
|  | Israel | 1 | 1.38 | [0.77; 2.46] | -- |  |
|  | Korea | 1 | 0.61 | [0.49; 0.75] | -- |  |

**Online Resource 13** Leave-one-out analysis for Bleeding events in patients with Chronic kidney disease.

| **Author** | **Effect** | **95% CI: Lower** | **95% CI: Upper** | **I^2** | **RStudent** | **DFFITS** | **Cook's D** | **Cov. R** | **Tau² (del)** | **QE (del)** | **Hat** | **Weight** | **Is Influential** |
| --- | --- | --- | --- | --- | --- | --- | --- | --- | --- | --- | --- | --- | --- |
| Omitting Herndon K, 2020 | 0.59 | 0.45 | 0.78 | 0.89 | -0.24 | 0.00 | 0.00 | 1.12 | 0.18 | 145.19 | 0.03 | 2.59 | no |
| Omitting Stanifer J, 2020 | 0.61 | 0.47 | 0.79 | 0.89 | -0.99 | -0.20 | 0.04 | 1.04 | 0.16 | 142.85 | 0.04 | 3.99 | no |
| Omitting Sy J, 2021 | 0.58 | 0.44 | 0.77 | 0.86 | 0.42 | 0.17 | 0.03 | 1.18 | 0.18 | 110.44 | 0.09 | 9.17 | no |
| Omitting Yao X, 2020 | 0.58 | 0.44 | 0.78 | 0.89 | 0.18 | 0.10 | 0.01 | 1.19 | 0.18 | 143.98 | 0.09 | 8.94 | no |
| Omitting Fu C, 2021 | 0.58 | 0.44 | 0.77 | 0.89 | 0.20 | 0.10 | 0.01 | 1.18 | 0.18 | 145.15 | 0.08 | 8.40 | no |
| Omitting Ahuja T, 2021 | 0.59 | 0.45 | 0.77 | 0.89 | 0.51 | 0.09 | 0.01 | 1.08 | 0.18 | 145.29 | 0.01 | 0.86 | no |
| Omitting Shin J, 2018 | 0.56 | 0.44 | 0.73 | 0.88 | 1.48 | 0.41 | 0.15 | 0.99 | 0.14 | 129.87 | 0.09 | 8.58 | no |
| Omitting Di Lullo L, 2018 | 0.62 | 0.49 | 0.78 | 0.89 | -2.16 | -0.39 | 0.15 | 0.76 | 0.11 | 139.29 | 0.02 | 1.87 | no |
| Omitting Schafer J, 2017 | 0.61 | 0.46 | 0.80 | 0.89 | -0.80 | -0.20 | 0.04 | 1.11 | 0.17 | 140.28 | 0.07 | 7.01 | no |
| Omitting Makani A, 2020 | 0.61 | 0.47 | 0.81 | 0.71 | -0.98 | -0.31 | 0.09 | 1.10 | 0.16 | 54.77 | 0.09 | 9.00 | no |
| Omitting Sarratt S, 2017 | 0.59 | 0.46 | 0.78 | 0.89 | -0.75 | -0.04 | 0.00 | 1.04 | 0.17 | 144.68 | 0.01 | 0.64 | no |
| Omitting Chashkina M, 2020 | 0.60 | 0.46 | 0.78 | 0.89 | -0.63 | -0.05 | 0.00 | 1.07 | 0.17 | 144.74 | 0.01 | 1.44 | no |
| Omitting Lee K, 2015 | 0.61 | 0.47 | 0.79 | 0.89 | -1.18 | -0.23 | 0.05 | 1.00 | 0.16 | 142.58 | 0.03 | 3.22 | no |
| Omitting Laugesen E, 2019 | 0.60 | 0.45 | 0.79 | 0.89 | -0.39 | -0.06 | 0.00 | 1.14 | 0.18 | 144.08 | 0.06 | 5.97 | no |
| Omitting Chang S, 2019 | 0.60 | 0.45 | 0.79 | 0.89 | -0.34 | -0.05 | 0.00 | 1.15 | 0.18 | 144.15 | 0.06 | 6.23 | no |
| Omitting Gurevitz C, 2021 | 0.57 | 0.45 | 0.73 | 0.89 | 1.85 | 0.34 | 0.10 | 0.87 | 0.13 | 139.83 | 0.06 | 5.99 | no |
| Omitting Kee K, 2023 | 0.59 | 0.44 | 0.78 | 0.89 | 0.07 | 0.07 | 0.00 | 1.19 | 0.18 | 144.13 | 0.09 | 8.57 | no |
| Omitting Hsu C, 2023 | 0.57 | 0.44 | 0.72 | 0.88 | 1.78 | 0.41 | 0.14 | 0.91 | 0.13 | 136.54 | 0.08 | 7.53 | no |

**Online Resource 14** Subgroup analysis for Mortality events in patients with Chronic kidney disease.

| **Variable** | **Subgroup** | **k** | **OR** | **95%-CI** | **I² (%)** | **p-value (Between Groups)** |
| --- | --- | --- | --- | --- | --- | --- |
| **Risk of Bias** | Low | 6 | 0.54 | [0.32; 0.93] | 97.5 | 0.01 |
|  | Some Concerns | 2 | 0.98 | [0.39; 2.50] | 0.0 |  |
| **Stage** | >4 | 2 | 0.98 | [0.39; 2.50] | 0.0 | 0.00 |
|  | All | 2 | 0.61 | [0.09; 3.98] | 66.1 |  |
|  | >3 | 4 | 0.51 | [0.18; 1.46] | 97.4 |  |
| **Study Design** | RCT | 2 | 0.98 | [0.39; 2.50] | 0.0 | 0.01 |
|  | Cohort | 6 | 0.54 | [0.32; 0.93] | 97.5 |  |
| **DOAC** | Apixaban | 2 | 0.74 | [0.02; 25.26] | 61.9 | 0.49 |
|  | DOACs | 4 | 0.54 | [0.19; 1.57] | 98.4 |  |
|  | Rivaroxaban | 2 | 0.52 | [0.17; 1.61] | 0.0 |  |
| **Follow-up** | 2 | 2 | 0.76 | [0.09; 6.31] | 40.1 | < 0.01 |
|  | 3.4 | 1 | 0.25 | [0.23; 0.28] | -- |  |
|  | 1.5 | 1 | 0.81 | [0.18; 3.59] | -- |  |
|  | Not specified | 2 | 0.50 | [0.23; 1.08] | 0.0 |  |
|  | 1 | 2 | 0.80 | [0.02; 40.70] | 85.0 |  |
| **Country** | United States | 3 | 0.54 | [0.09; 3.19] | 98.4 | 0.01 |
|  | Russia | 1 | 0.81 | [0.18; 3.59] | -- |  |
|  | South Korea | 1 | 0.37 | [0.08; 1.68] | -- |  |
|  | Denmark | 1 | 1.07 | [0.82; 1.40] | -- |  |
|  | Israel | 1 | 0.58 | [0.39; 0.85] | -- |  |
|  | Korea | 1 | 0.51 | [0.38; 0.69] | -- |  |

**Online Resource 15** Leave-one-out analysis for Mortality events in patients with Chronic kidney disease.

| **Author** | **Effect** | **95% CI: Lower** | **95% CI: Upper** | **I^2** | **RStudent** | **DFFITS** | **Cook's D** | **Cov. R** | **Tau² (del)** | **QE (del)** | **Hat** | **Weight** | **Is Influential** |
| --- | --- | --- | --- | --- | --- | --- | --- | --- | --- | --- | --- | --- | --- |
| Omitting Stanifer J, 2020 | 0.55 | 0.35 | 0.89 | 0.97 | 1.09 | 0.41 | 0.16 | 1.11 | 0.19 | 201.42 | 0.12 | 12.15 | no |
| Omitting Yao X, 2020 | 0.58 | 0.35 | 0.98 | 0.96 | 0.32 | 0.13 | 0.02 | 1.36 | 0.22 | 145.27 | 0.17 | 16.63 | no |
| Omitting Makani A, 2020 | 0.72 | 0.53 | 0.97 | 0.66 | -3.95 | -1.92 | 1.01 | 0.45 | 0.05 | 17.46 | 0.17 | 17.11 | yes |
| Omitting Chashkina M, 2020 | 0.59 | 0.36 | 0.96 | 0.97 | 0.35 | 0.07 | 0.00 | 1.19 | 0.22 | 213.89 | 0.04 | 4.32 | no |
| Omitting Lee K, 2015 | 0.61 | 0.38 | 0.99 | 0.97 | -0.54 | -0.13 | 0.02 | 1.16 | 0.22 | 215.05 | 0.04 | 4.25 | no |
| Omitting Laugesen E, 2019 | 0.53 | 0.34 | 0.83 | 0.96 | 1.54 | 0.70 | 0.41 | 1.01 | 0.16 | 144.31 | 0.16 | 15.77 | no |
| Omitting Gurevitz C, 2021 | 0.60 | 0.36 | 1.01 | 0.97 | -0.07 | -0.04 | 0.00 | 1.35 | 0.23 | 208.92 | 0.14 | 14.36 | no |
| Omitting Kee K, 2023 | 0.61 | 0.37 | 1.03 | 0.97 | -0.35 | -0.16 | 0.03 | 1.34 | 0.22 | 209.19 | 0.15 | 15.41 | no |
